# Ketogenic Diet in Neuromuscular and Neurodegenerative Diseases

**DOI:** 10.1155/2014/474296

**Published:** 2014-07-03

**Authors:** Antonio Paoli, Antonino Bianco, Ernesto Damiani, Gerardo Bosco

**Affiliations:** ^1^Department of Biomedical Sciences, University of Padova, Via Marzolo 3, 35031 Padova, Italy; ^2^Sport and Exercise Sciences Research Unit, University of Palermo, Via Eleonora Duse 2, 90146 Palermo, Italy

## Abstract

An increasing number of data demonstrate the utility of ketogenic diets in a variety of metabolic diseases as obesity, metabolic syndrome, and diabetes. In regard to neurological disorders, ketogenic diet is recognized as an effective treatment for pharmacoresistant epilepsy but emerging data suggests that ketogenic diet could be also useful in amyotrophic lateral sclerosis, Alzheimer, Parkinson's disease, and some mitochondriopathies. Although these diseases have different pathogenesis and features, there are some common mechanisms that could explain the effects of ketogenic diets. These mechanisms are to provide an efficient source of energy for the treatment of certain types of neurodegenerative diseases characterized by focal brain hypometabolism; to decrease the oxidative damage associated with various kinds of metabolic stress; to increase the mitochondrial biogenesis pathways; and to take advantage of the capacity of ketones to bypass the defect in complex I activity implicated in some neurological diseases. These mechanisms will be discussed in this review.

## 1. Introduction

It is known that single nutrients may exert positive effects on skeletal muscle health and moreover a combination of nutrients can attenuate signs and symptoms of some neuromuscular diseases. On the other side it is also known that the effects of dieting on health are related to the general ratio of the different macro- and micronutrients rather than each single component. From this point of view great interest has raised in the last years over ketogenic diet (KD).

Since the third decade of the XX century KD is used to treat patients with pharmacological resistance to epilepsy [1–3]. In more recent periods, KD has also been claimed to be useful in other totally different diseases as obesity [4], PCOS [5], cancer [1, 6, 7], diabetes [8], or other pathological conditions [9–11]. Whilst many studies have pointed out potentially positive effects of KDs on many neurological and neuromuscular diseases only few researches have investigated the mechanisms of this promising nutritional approach [12]. The aim of our review is to discuss the role of KDs in selected diseases that affect nervous system with implications in muscular function.

## 2. Inside the Ketogenic Diet

**Figure 1:**
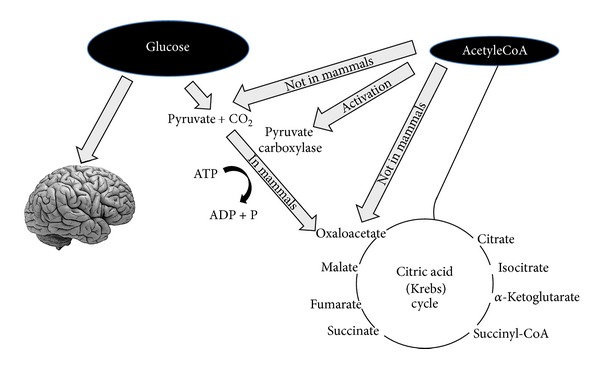
Glucose is necessary not only to supply energy for the central nervous system but also to produce pyruvate that can be transformed into oxaloacetate. Oxaloacetate must be maintained at a level sufficient to allow citric acid cycle function (i.e., the condensation between acetyl-CoA and oxaloacetate). Oxaloacetate is unstable and must be refurnished (this kind of reactions is called anaplerotic). The main way to produce oxaloacetate is from pyruvate that derives from glucose. In mammals pyruvate cannot be produced from acetyl-CoA as shown in the figure.

After a few days of fasting or a drastic reduction in carbohydrate from the diet (below 20 g per day), glucose reserves become insufficient both (1) for normal fat oxidation through the supply of oxaloacetate in the Krebs cycle and (2) for the supply of glucose to the CNS (central nervous system) [13, 14] (Figure 1).

Regarding point (1) oxaloacetate is relatively unstable at body temperature and cannot be accumulated in the mitochondrial matrix; thus, in this “glucose deprivation” condition, it is necessary to supply oxaloacetate for an efficient functioning of the tricarboxylic acid cycle. Oxaloacetate is supplied via the anaplerotic cycle that synthesizes it from glucose through ATP dependent carboxylation of pyruvic acid by pyruvate carboxylase [15].

Regarding point (2), since the CNS cannot use fatty acids (FFA) as an energy source (FFA cannot cross the blood-brain barrier), it normally utilizes glucose. After 3-4 days without any carbohydrate intake the CNS must find alternative energy sources as demonstrated by the classic experiments by Felig et al. [13, 14, 16, 17]. This alternative energy source is the ketones bodies (KBs): acetoacetate (AcAc), 3-hydroxybutyrate (3HB), and acetone [18], derived from the overproduction of acetyl-CoA without a concomitant production of an adequate amount of oxaloacetic acid. This process is called ketogenesis and principally occurs in the mitochondrial matrix in the liver [19]. It is important to underline that the liver produces KBs but is also unable to use them because of the absence of the succinyl-CoA: 3-CoA transferase (SCOT) enzyme required to convert acetoacetate into acetoacetyl-CoA [18].

**Figure 2:**
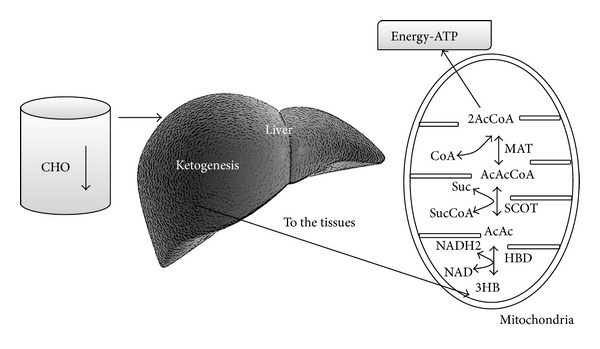
A reduced availability of dietary carbohydrates leads to an increased liver production of KBs. The liver cannot utilize KBs because it lacks the mitochondrial enzyme succinyl-CoA: 3-ketoacid (oxoacid) CoA transferase (SCOT) necessary for activation of acetoacetate to acetoacetyl-CoA. KBs are utilized by tissues, in particular by brain. KBs enter the citric acid cycle after being converted to acetyl-CoA by succinyl-CoA: 3-CoA transferase (SCOT) and methylacetoacetyl-CoA thiolase (MAT).

The main ketone body produced in the liver is acetoacetate but the primary circulating ketone is 3-hydroxybutyrate. Under normal conditions the production of free acetoacetic acid is negligible and can be metabolised by various tissues such as skeletal muscle and the heart. In conditions of overproduction of acetoacetic acid this accumulates above normal levels and part is converted to the other two ketone bodies. High level of KBs in the blood and their elimination via urine cause ketonemia and ketonuria. Under normal conditions the concentration of KBs is usually very low (<0.3 mmol/L) compared to glucose (approx. 4 mmol/L) [20, 21]. Once KBs reach a concentration of about 4 mmol/L (which is close to the k_M_ for the monocarboxylate transporter [22]) they start to be utilised as energy source by the CNS [21]. KBs are used by tissues as a source of energy [19, 21, 23] through a pathway that firstly converts 3HB back to AcAc which is then transformed into acetoacetyl-CoA. The latter is finally divided into two molecules of acetyl-CoA, which are subsequently used in the Krebs cycle (Figure 2). It is interesting to note that, compared to glucose, the KBs are able to produce a higher quantity of energy due to the changes in mitochondrial ATP production that they induce [21, 24, 25].

Another point to highlight is, as shown in Table 1, that glycaemia, even though reduced, remains within physiological levels [26, 27], due to main two sources: (1) glucogenic amino acids and (2) glycerol liberated via lysis from triglycerides [28, 29]. During physiological ketosis (fast or very low calorie KD) ketonemia reaches maximum levels of 7/8 mmol/L with no changes in pH while in uncontrolled diabetic ketoacidosis this can exceed 20 mmol/L with a concomitant lowering of blood pH [16, 30] (Table 1). Blood levels of KBs in healthy people do not exceed 8 mmol/L because the central nervous system (CNS) efficiently uses these molecules as energy supply in place of glucose [16].

## 3. Ketogenic Diet Simulates Fasting and Its Molecular Effects

Traditionally, physicians are afraid of ketosis because they associate the severe hyperketonemia that results from insulin deficiency and that can lead to severe acidosis and death in individuals with type 1 diabetes with physiological ketosis resulting from fasting or KD. Hans Krebs was the first who used the term “physiological ketosis” to distinguish the mild (≅8 mmol/L of KBs) ketosis of starving or KD from the “pathological ketoacidosis” of metabolically unbalanced diabetes [31].

Despite the concerns of many physicians, it is known that no other species, except humans, are evolutionarily adapted to chronic undernutrition as a consequence of ecologically dictated dietary restrictions [32]. The periods of fasting or undernutrition are themselves ketogenic [23] during which the concentrations of insulin and glucose decrease while those of glucagon increase with the attempt to maintain normal blood glucose levels. When the body passes from a condition of food abundance to one of deprivation (or else via KD simulated deprivation), there is, with a slight delay, an increase in the concentration of FFA and KBs in the blood. Thus, from this point of view a KD could be compared to a caloric restriction, undernutrition, or fasting. This manipulation of nutrients, both in quantity and quality, seems to act both on blood glucose and KBs and also has the capacity to promote changes in metabolic pathways and cellular processes such as stress resistance and autophagy. KDs can also act in a way similar to caloric restriction (CR) on AMPK and SIRT-1 [33]. Thus, to understand the complex effects of KDs we have to take into account these molecular and intracellular pathways. PGC1*α* is activated in the phosphorylated state. Once phosphorylated, PGC1*α* translocates from the cytosol to the nucleus, where it promotes the transcription of genes involved in fatty acid transport, fat oxidation, and oxidative phosphorylation [34]. PGC1*α* could be phosphorylated through several different pathways, among them AMPK, calcium-calmodulin-dependent protein-kinase, and p38 mitogen-activated protein kinase pathways [35]. PGC1*α* can also be activated by a SIRT1-mediated deacetylation [36]. AMPK can exert its action both via phosphorylation of PGC1*α* or in a direct manner. AMPK activation promotes enhanced expression of skeletal muscle oxidative-related enzymes, proteins, and metabolism, which are consistent with the findings that obese skeletal muscles are less oxidative and have lower AMPK activation (during fasting conditions). At the same time, AMPK activation also inhibits mTOR signalling. However, it seems counterintuitive to inhibit an important growth-mediated pathway (i.e., mTOR), regulating muscle mass, so that skeletal muscles can grow [37]. Thus, nutrients manipulation may influence these pathways; for example, relative deficiency in carbohydrate availability has been demonstrated to be a stimulus* in vivo* for activation of AMPK and SIRT-1, increasing phosphorylation of AMPK and deacetylation of PGC1*α* in skeletal muscle without affecting the total amount of AMPK, PGC1*α*, or SIRT 1 [38]. These mechanisms appear to be activated just after few hours (5 hours) of starvation in mice [39] whilst no data are available on KD and humans. Once activated, SIRT1 and AMPK produce beneficial effects on glucose homeostasis and insulin action [40].

## 4. Ketogenic Diet and Amyotrophic Lateral Sclerosis

Amyotrophic lateral sclerosis (ALS) is a progressive neurodegenerative disorder that affects spinal and cortical motor neurons, leading to progressive weakness and loss of skeletal muscle. Affected subjects die within 2 to 5 years of symptom onset. Death usually occurs from respiratory paralysis. At the moment, there are no effective treatments for ALS, and the only US FDA approved pharmacological therapy is limited to riluzole that causes only a modest decrease of the disease progression and increases survival by only 2 to 3 months [41]. The causes of ALS are complex and multifactorial, embracing genetic and environmental factors: excessive oxidative damage, neurofilament accumulation, excitotoxicity, and mitochondrial membrane dysfunction are some of the supposed causes [42–44].

Due to its multifactorial origins, specific targets of treatment have not been identified yet and, unfortunately, an effective therapy is still missing. As other neurodegenerative disorders the probable mitochondrial involvement makes the KD a promising synergic tool for the treatment of ALS [45]. In about 10% of patients with ALS, this is an inherited disorder (familial amyotrophic sclerosis FALS) and in 20% of these subjects there is a mutation in the gene encoding the enzyme Cu/Zn superoxide dismutase 1 (SOD1) [46]. This mutation is linked to mitochondrial activity; it is in fact the mutant SOD1 that has been localized in the mitochondria binding bcl2 (a cell antiapoptotic protein) [47]. Moreover decreased mitochondrial complex I activity has been measured in skeletal muscle and spinal cord of ALS patients [48]. Results show that KB can act on mitochondrial function, restoring, for example, complex I function, after a pharmacological blocking. Moreover, in cultured neurons treated with pharmacologic agents blocking complex I, an addition of KB restores the function of the complex [49].

Recently, researchers have demonstrated that in an ALS mouse model (a KO mouse for the copper/zinc SOD1-G93A) the administration of KD led to a higher motor neuron survival and an improvement in motor function compared to KO mice without KD [50]. Other interesting studies have reported that a supplementation with a precursor of KB (caprylic acid) improved mitochondrial function and motor neuron count in an ASL mouse model [51]. Authors explained these results through a neuroprotective effect of DHB. Moreover, they suggested that the hyperketonemia might improve the mitochondrial defects by increasing mitochondrial function and ATP production (measured in purified mitochondria from an ASL mouse model) even if in both studies there were no significant increases in survivals. It is important to underline that during the KD the percentage of dietary fat was very high (60%) and this could explain the measured improvements. As a matter of fact, cholesterol and phospholipids are essential for axonal membrane health and for peripheral nerve membrane injury repairs, in particular low-density lipoproteins [52]. Interestingly, there are some epidemiological data demonstrating that hyperlipidaemia is a significant prognostic factor for survivals in patients with ASL [53], but these results were not confirmed by Paganoni et al. [54] who showed a “U-”shaped association between BMI and mortality, with the highest survival in subjects with higher BMI (30–35); in this study dyslipidemia is not an independent predictor of survival in ALS. Wills et al. [55] have recently showed that patients which received a high caloric/high carbohydrate enteral nutrition had a smaller total number of adverse events and deaths than those of the high fat/high calorie group or the control group. These apparently contradictory results depict a complex scenario in ALS. However there are some common features: a higher caloric intake seems to improve survival in ALS patients, even though no conclusive relationships have been found between cholesterol and improved conditions. It is known that endogenous cholesterol production is enhanced by insulin and reduced by exogenous cholesterol [4]; thus a high carbohydrate diet could have been useful to improve cholesterol production. It could be hypothesised that the positive effects of a high caloric high carbohydrate diet could be used in alternating periods with a high fat (high butter [56]) KD in some types of ALS (SOD1) but not in those linked to RNA processing perturbations (TDP43, FUS, and C9orf72). More CRTs are needed to investigate the role of nutrition, and more in detail of KD, in ALS therapy.

## 5. Mitochondrial Disorders and Ketogenic Diet

In the previous paragraph we have discussed the role of mitochondria in a neurological disease like ALS. There is an increasing amount of evidence that KD can improve mitochondrial functioning and stimulate mitochondriogenesis [57–60]. As stated by Wallace and colleagues, “Ironically, one of the oldest therapeutic approaches—fasting and the ketogenic diet—remains the most promising treatment for mitochondrial defects” [61]. As a matter of fact, even though KD as therapeutic tool is known since the 20's of XX century its effects on mitochondria are a, relatively, recent finding. Some mitochondrial defects may cause seizures with different epileptic phenotypes [62]. There are some encouraging data about the effects of KDs in mitochondriopathies. Kang et al. [63] showed that a KD could be a safe and effective therapy that reduces seizures in children with intractable epilepsy and various respiratory complex defects (complex I, II, IV, or combined).

Ahola-Erkkilä et al. [64] have treated a mouse model for late-onset mitochondrial myopathy that is known to cause in humans autosomal dominant progressive external ophthalmoplegia, with generalized muscle weakness, accumulation of generalized mtDNA deletions, and cytochrome c oxidase negative muscle fibers with a KD. The KD decreased the amount of cytochrome c oxidase negative muscle fibers and prevented the formation of the mitochondrial ultrastructural abnormalities in the muscle. The diet cured most of the metabolic and lipidomic anomalies not through an action on mtDNA but inducing mitochondrial biogenesis. Nevertheless, we have to consider the two sides of the same coin: even though the KD might be a therapeutic tool in many mitochondrial-based diseases it is contraindicated in several metabolic disorders. Patients with fat metabolism disorder might undergo severe catabolic crisis.

Inborn errors in the enzymes involved in lipid metabolism: from mitochondrial membrane long-chain fatty acids transport mechanism to beta-oxidation and Krebs cycle could be potentially fatal during fasting or KDs. Thus, carnitine deficiency, carnitine palmitoyltransferase (CPT) I or II deficiency, carnitine translocase deficiency, b-oxidation defects, or pyruvate carboxylase deficiency should be screened before initiating the KD treatment. Moreover, KD can exacerbate acute intermittent porphyria in affected subjects [65].

## 6. Alzheimer's Disease and Ketogenic Diet

Alzheimer's disease (AD) is the most prevalent neurodegenerative disease and the leading cause of dementia among the aged population. AD symptoms are in general a cognitive impairment with progressive memory deficits and personality changes. The causes of such cognitive decline can be attributed to a progressive synaptic dysfunction and the subsequent loss of neurons; this loss seems to be located in many vulnerable regions of the brain: mainly neocortex, limbic system, and the subcortical regions [66]. The hippocampus is a specific target of KD; McDaniel et al. demonstrated that in a kainic acid- (KA-) induced status epilepticus rat model, the KD inhibited mTOR pathway signalling in the brain preventing late hippocampal mTOR activation after KA-induced status epilepticus [59]. It is important to underline that neurons in the hippocampus play critical roles during learning and memorization and are particularly vulnerable to dysfunction and degeneration in AD. AD has been categorized into two major forms: familial AD (FAD) and sporadic AD (SAD) or late onset age-related AD (LOAD); the latter is the leading cause of dementia, accounting for more than half of all cases. Whilst quite all FAD cases can be attributed to mutations in three genes (amyloid precursor protein APP, presenilin 1 PSEN1, and presenilin 2 PSEN2 [67]), the exact etiology of SAD is not still completely understood. It is well known that age is the greatest risk factor (AD increases exponentially with age in people aged 65 or older) [68], together with some other factors as (1) allelic variations in apolipoprotein E (Apo E), (2) degeneration of anatomical pathways, (3) mitochondrial dysfunction, (4) compromised blood-brain barrier, (5) immune system dysfunction, (6) infectious agents and other environmental factors such as exposure to aluminium, (7) repeated head injury, and (8) malnutrition [69].

As in other chronic diseases, treatments for AD can be divided into two categories: (A) symptomatic treatments (that offer temporary amelioration of symptoms without modifying disease progression over years) and (B) treatments that are potentially able to modify disease's history (slowing or halting the decline of cognitive functions over years). Despite some FDA approved drugs like acetyl cholinesterase inhibitors and memantine (a glutamate antagonist used to ameliorate behavioural symptoms, in the moderate phase of the disease) currently no effective treatment exists to prevent, modify, or stop AD. Most of the approved drugs for treatments only offer a moderate symptomatic effect [70, 71]. As for other diseases the development of effective treatments is hindered by the not complete knowledge of AD etiology [71] even now that the “amyloid cascade” hypothesis has been extensively studied. This pathogenetic hypothesis is based on the neurotoxic characteristics of *β*-amyloid (A*β*) and on its accumulation related initiation of a cascade of neurotoxic events, including not only the formation of well-known neurofibrillary tangles (NFT) but also chronic inflammatory responses, an increase in oxidative stress, and in conclusion a mitochondrial dysfunction [71]. The two major lesions in AD are caused by distinct proteins, tau in the case of the neurofibrillary tangles and amyloid *β*-protein in the case of amyloid plaques.

Nevertheless, as aforementioned, there is no unified etiopathogenic mechanism for both FAD and SAD. In the latter there are data suggesting that the deposition of amyloid *β*-protein and NFTs act together inducing a decline in mitochondrial function and alter brain metabolic activity, all relate aging processes. In consideration of the strong link between aging process and AD and of the positive effects of KD in ageing brains [72], the multifaceted nature of AD that includes mitochondrial and metabolic dysfunctions suggests that there could be a rationale for the use of KD in these patients [73, 74]. For example, an* in vitro* study has demonstrated that addition of KB (beta-hydroxybutyrate) protects the hippocampal neurons from A*β* toxicity; this suggests possible therapeutic roles for KD on mitochondrial defects related to AD [75]. Animal studies though showed contrasting results: Van der Auwera et al. [76] demonstrated a decrease of A*β* in the brain of young transgenic AD mice overexpressing the London APP mutation fed with KD for 1.5 months whilst on aged canines the effect of KD on A*β* seemed to be limited to the parietal lobe of the brain [77]. It was also demonstrated that long-term (8 months) feeding of a ketone ester in middle-aged mice (8.5 months old) improved cognition and ameliorated A*β* and tau pathology [75]. Beckett et al. [78] demonstrated that AD mice model fed with a high-fat, low-carbohydrate ketogenic diet showed an improved motor function without changes in A*β*. The contradictory results between animal studies could be due to the age of the animals: mice are in most cases young or middle aged but there are recurrent metabolic alterations mainly expressing in the elderly. For example, AD is also associated with metabolic dysregulation and insulin resistance [79]. Many researchers have demonstrated that KD could significantly improve glucose homeostasis, reducing metabolic dysregulation and insulin resistance [80–82]. There is another pathophysiological mechanism ascribed to an altered mitochondrial function and glucose metabolism in AD: the accumulation of advanced glycation end products (AGEs) [83]. Despite the fact that the accumulation of AGEs in cells and tissues is a normal feature of aging, this process is accelerated in AD. AGEs can be also found in amyloid plaques and neurofibrillary tangles. Increases of AGEs can clearly explain many neuropathological aspects of AD (protein crosslinking, glial induction of oxidative stress, and neuronal cell death). We can speculate that the neuroprotective effects of KDs and the KDs' related reduction of glycaemia could also improve these features in AD. Another intriguing hypothesis, in our opinion, is the putative effects of a KD on mitochondriogenesis together with the improvement of mitochondria machinery [61, 72, 74, 84–86].

As previously stated, mitochondrial dysfunction has been hypothesised to be implicated in the aetiology of AD [72]. A reduction in neuronal and glial mitochondrial metabolism was shown in elderly compared to healthy young subjects [87]. This dysfunction, related to diminished energy production from mitochondrial glucose/pyruvate oxidation, could enhance the pathologic deposition of A*β* and tau. This impaired mitochondrial function could be represented by an increased superoxide production with oxidative damage, decrease in oxidative phosphorylation, and, consequently, impairment of the mitochondrial electron transport chain. Other glucose metabolic impairments in specific zones of the brain, which are characteristic of AD, are related to mitochondrial dysfunction [88]. It is interesting to note that earlier reduced glucose utilization could be detected by FDG-PET in cognition-related brain sites in subjects with familiar history of AD [89]. It is possible that a reduced level of brain glucose utilization may contribute to the development of AD neuropathology. As suggested by Vanitallie and coworkers an early impairment in brain glucose metabolism can be detected before any measurable cognitive decline [90]. Other evidence supports this theory, such as a reduced concentration of glucose transporters (GLUT 1 and 2 but also the neuronal glucose transporter GLUT 3). It is shown that in the brain there is abnormal hyperphosphorylation of tau in AD related to this phenomenon [91]. Considering the shift in the brain's metabolism from glucose to ketones, during a KD [17], shift that is useful in glucose transporter type I deficiency syndrome [92], KD might be a therapy for neuronal degeneration related to GLUT deficiency in AD [73]. The brain in the AD also seems to be able to use KB as a fuel, when glucose utilization is impaired. A study has demonstrated that a supplementation with medium chain triglycerides that induce an increase of KB improved performance in the AD Assessment Cognitive Scale with a direct correlation between ketone concentration and cognitive improvement [93].

Finally, even though there are no direct or strong evidence of the usefulness of KD in humans, this nutritional approach appears promising and so deserves further clinical extensive trials.

## 7. Parkinson Disease and Ketogenic Diet

The pathogenesis of sporadic Parkinson disease (PD) remains unresolved, but numerous studies suggest that the primary cause is excitotoxic degeneration of dopaminergic neurons in the substantia nigra, leading to abnormalities of movement, and to an increasing extent in cognition and other cortical function disorders. It has been suggested that an impairment of mitochondrial function involving the substantia nigra plays an important contributory role in PD beginning and progression [94]. For example, Kashiwaya et al. used a heroin analogue 1-methyl-4-phenylpyridinium, MPP(+), that produces death of dopaminergic substantia nigra cells by inhibiting the mitochondrial NADH dehydrogenase multienzyme complex, producing a syndrome similar to Parkinson's disease in cultured mesencephalic neurons. *β*-Hydroxybutyrate protected these neurons from MPP(+) toxicity neurodegeneration [74]. In animal models, 1-methyl-4-phenol-1,2,5,6-tetrahydropyridine (MPTP) is used to produce selective destruction of dopaminergic neurons in the substantia nigra that mimics human Parkinson's disease-like syndrome. As for other, abovementioned, diseases the positive effects of KD on mitochondrial function could be a key factor in the utilization of such diet as ketones may bypass the defect in complex I activity implicated in PD. Infusion of *β*-hydroxybutyric acid in mouse protects from ageing the dopaminergic neurodegeneration and motor deficits induced by MPTP [49]. Moreover KD protected dopaminergic neurons of the substantia nigra against 6-hydroxydopamine neurotoxicity in a rat model of Parkinson disease [95]. VanItaille et al. [96] demonstrated that in humans, able to prepare a “hyperketogenic” diet at home and adhere to it for 28 days, the high level of KB was related to an improvement in the Unified Parkinson's Disease Rating Scale scores.

## 8. Glycogenoses and Ketogenic Diet

Glycogenoses (glycogen storage diseases, GSD) are a group of inherited disorders due to enzyme defects, affecting glycogen metabolism and leading to intracellular accumulation of glycogen of normal or abnormal structure in a variety of cell types. Classically, GSD were numbered I to VIII, according to the chronology of their discovery and the specific enzyme defect [97]. In recent years, other primary glycogenoses (GSD 0, GSD IX to XV) were identified [98]. GSD are transmitted as autosomal recessive, with the exception of GSD VIII (also classified as IXa), that is X-linked. From a functional point of view, GSD I, III, IV, VI, and VIII/IXa can be grouped as hepatic GSD [99], since defective enzymes are mostly expressed in liver cells. Given the central role of liver in regulation of glycaemia through glycogenolysis, it is not surprising that hypoglycemia is the main manifestation of hepatic GSD [97, 100]. This, in turn, causes neurological symptoms ranging from convulsion to seizure, particularly in the early years of life. In the long term, recurrent severe hypoglycemia may cause brain damage, particularly in GSD I (von Gierke disease, deficiency of G-6-P phosphatase), the most frequent hepatic GSD. Up to date, treatment of hepatic GSD is based on dietary therapy to prevent hypoglycemia, feeding patients with foods rich in starches during the night and the day [100, 101]. The scientific rationale for a potential use of a ketogenic diet (KD) stems from the early observation that symptoms related to hypoglycemia improved with age in GSD [102], as well as GSD III [100] patients. It is well known that this adaptation occurs in the brain during starvation as well as during fever [102]. This observation was classically interpreted as a consequence of adaptations taking place in the brain and allowing an increased use of ketone bodies as fuel substrates alternative to glucose. The same mechanism has been put forward for explaining the effect of calorie restriction [100] that also results in low blood glucose levels. The mechanistic interpretation would be that KD would increase the utilization in the brain of pathways in energy metabolism independent of glycogen breakdown. Based on these considerations, KD has been effectively used in the treatment of muscular GSD V (McArdle disease) [103, 104]. The anticonvulsing effects of the KD are well established, even though the mechanisms are not fully clarified yet [105]. The potential use of KD in pathological conditions characterized by chronic hypoglycemia is further strengthened by the fact that KD is the gold standard in the treatment of GLUT1 deficiency syndrome [106], which can be considered a metabolic phenocopy of hepatic GSD, since blood glucose cannot be transported into neuron. Finally, a recent study [107] suggested that KD might be successfully used in treatment of severe cardiomyopathy complicating the muscular form of GSD III. Altogether, these observations might encourage further studies on the use of KD in treatment of selected forms of GSD.

## 9. Conclusions

The peculiar metabolic state induced by a KD has been widely investigated in the last years. The increase of KBs concentration, the reduction of blood glucose together with the involvement of many important pathways (e.g., IGF-1/AKT/mTor, AMPK/PGC1*α*) has shown to be a potential therapeutic weapon against many neurological and neuromuscular diseases.

Although these studies provide a theoretical basis for the effect of KDs on a number of neuromuscular diseases, several important hurdles remain before these findings can be applied widely to clinical practice or public health efforts. First, little is known about the precise mechanism of KD action on neuromuscular diseases and, second, long term effects of this kind of diet should be investigated in these patients.

Despite the fact that we only have preliminary evidence based mostly on animal models, most available data sets indicate that the putative mechanism of KDs on some neurological and neuromuscular diseases could be as follows.

1. Provide an efficient source of energy for the treatment of certain types of neurodegenerative diseases characterized by focal brain hypometabolism such as Parkinson and Alzheimer diseases. Neuronal cells are capable of metabolizing KBs even in the presence of a deficiency of glucose. Ketones can increase the Δ*G* of ATP hydrolysis and provide a source of cytoplasmic acetyl-CoA that can blunt the lowering of acetyl choline characteristic of Alzheimer's brains.
2. Decrease the oxidative damage associated with various kinds of metabolic stress. If compared with glucose metabolism, ketones generate lower levels of oxidative stress in the brain together with a greater cellular energy output and antioxidant capacity. Moreover, ketosis can increase glutathione peroxidase in hippocampal cells and in general decreases mitochondrial ROS production.
3. Increase the mitochondrial biogenesis pathways (through activation of AMPK and PGC 1 *α* pathway). The improvement of mitochondrial pathways can help to improve brain and neuronal metabolism.
4. Allow ketones to bypass the defect in mitochondrial complex I activity founded in skeletal muscle and spinal cord of ALS. In cultured neurons treated with pharmacologic agents blocking complex I, an addition of KB restores the function of the complex.
5. Decrease the amount of cytochrome-c oxidase negative muscle fibers in some mitochondrial myopathy and prevent the formation of the mitochondrial ultrastructural abnormalities in the muscle.

In conclusion, we believe that KD should be studied in more depth for its encouraging prospective as a therapy for many neuromuscular and neurodegenerative diseases.

## Figures and Tables

**Table 1:** Blood levels during a normal diet, ketogenic diet (i.e., <20 grams of carbohydrates per day), and diabetic ketoacidosis [10].

Blood levels	Normal diet	Ketogenic diet	Diabetic ketoacidosis
Glucose (mg/dL)	80–120	65–80	**>300**
Insulin (*μ*U/L)	6–23	6.6–9.4	≅**0**
KBs conc. (mmol/L)	0.1	7/8	**>25**
pH	7.4	7.4	**<7.3**
